# Supplementary figures and images for: PERK modulation, with GSK2606414, Sephin1 or salubrinal, failed to produce therapeutic benefits in the SOD1G93A mouse model of ALS
Source: PLoS One. 2024 Feb 15;19(2):e0292190. doi: 10.1371/journal.pone.0292190 (PMC10868768; doi:10.1371/journal.pone.0292190)

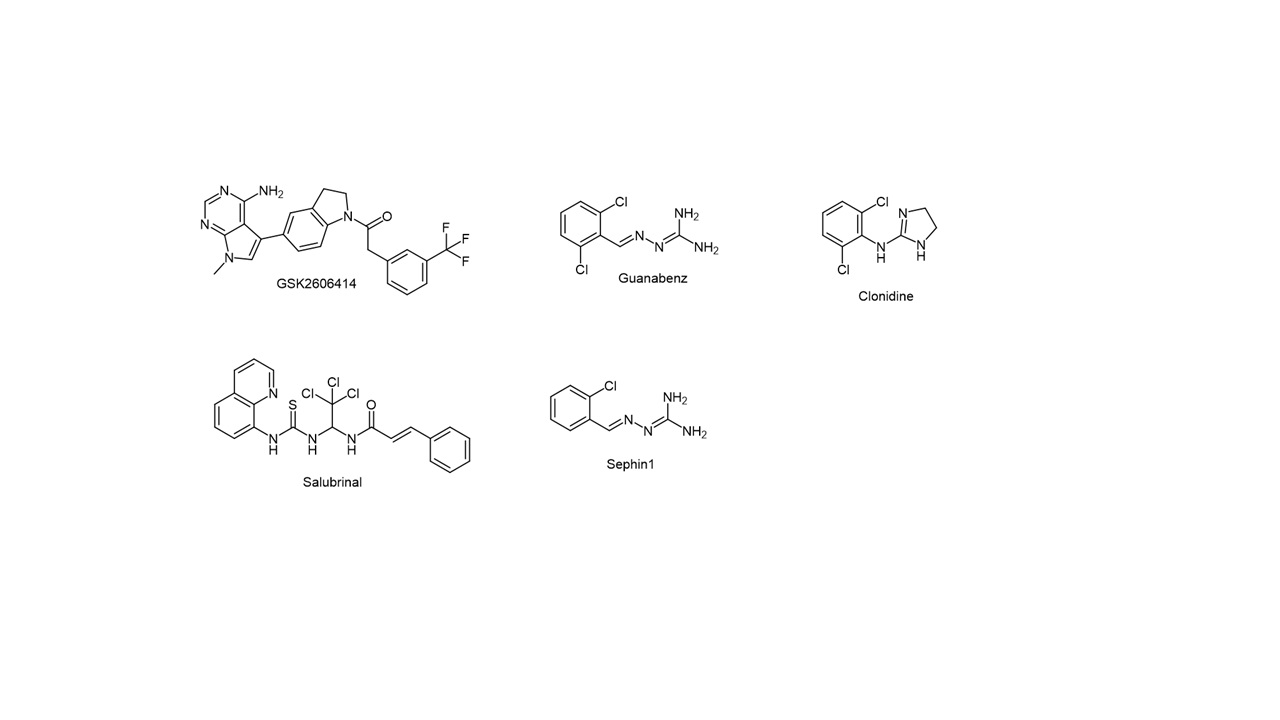

Supplement: S1 Fig — (TIF) [file pone.0292190.s001.tif]

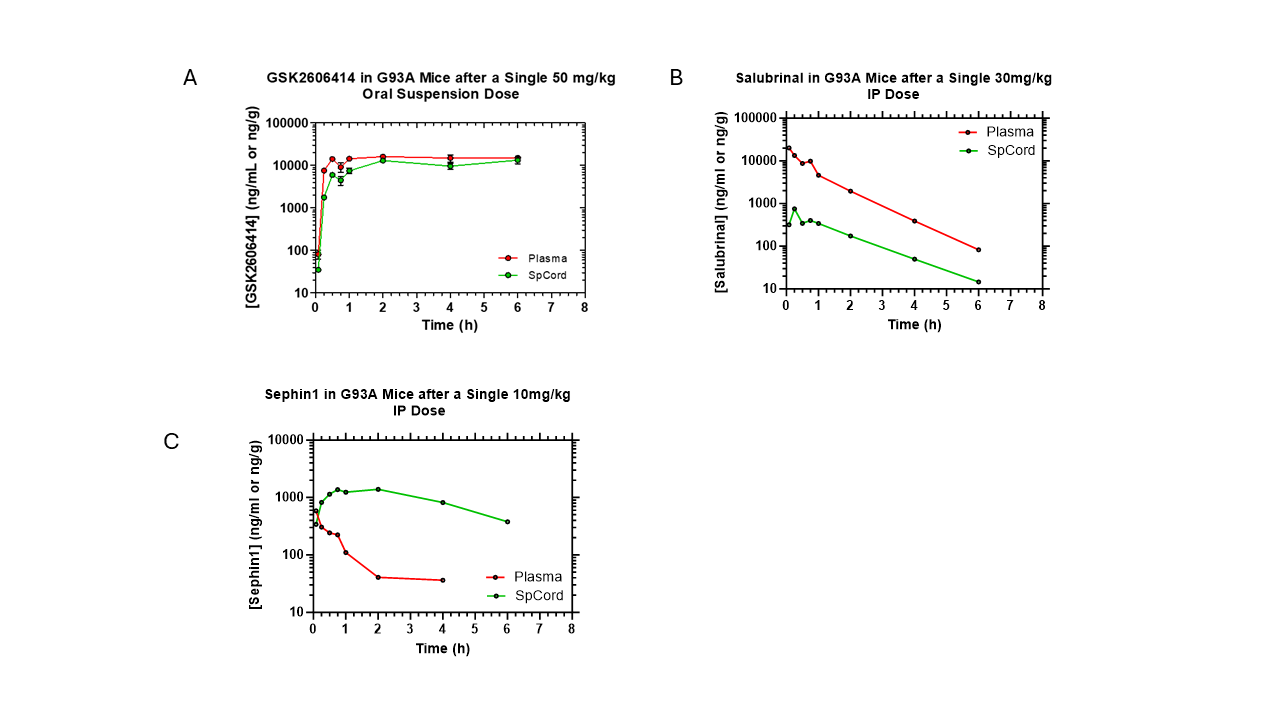

Supplement: S2 Fig — Plasma and spinal cord drug levels in SOD1G93A mice following a single bolus injection of A) GSK260641410 at a dose of 50mg/kg via PO administration, B) Salubrinal at a dose of 30mg/kg via IP administration, and C) Sephin1 at a dose of 10mg/kg via IP administration. Tissue samples were obtained at various time points (5, 15, 30, 45 minutes, and 1, 2, 4, 6 hours post-administration) and processed for analysis using LC-MS/MS. Blood samples were collected through intracardial puncture, followed by centrifugation (3000 g for 10 min) to obtain plasma. Spinal cords were collected by flushing the vertebral column with saline after a gravity perfusion using 5ml of saline. The values shown represent the mean of data obtained from three mice at each specified time point. (TIF) [file pone.0292190.s002.tif]

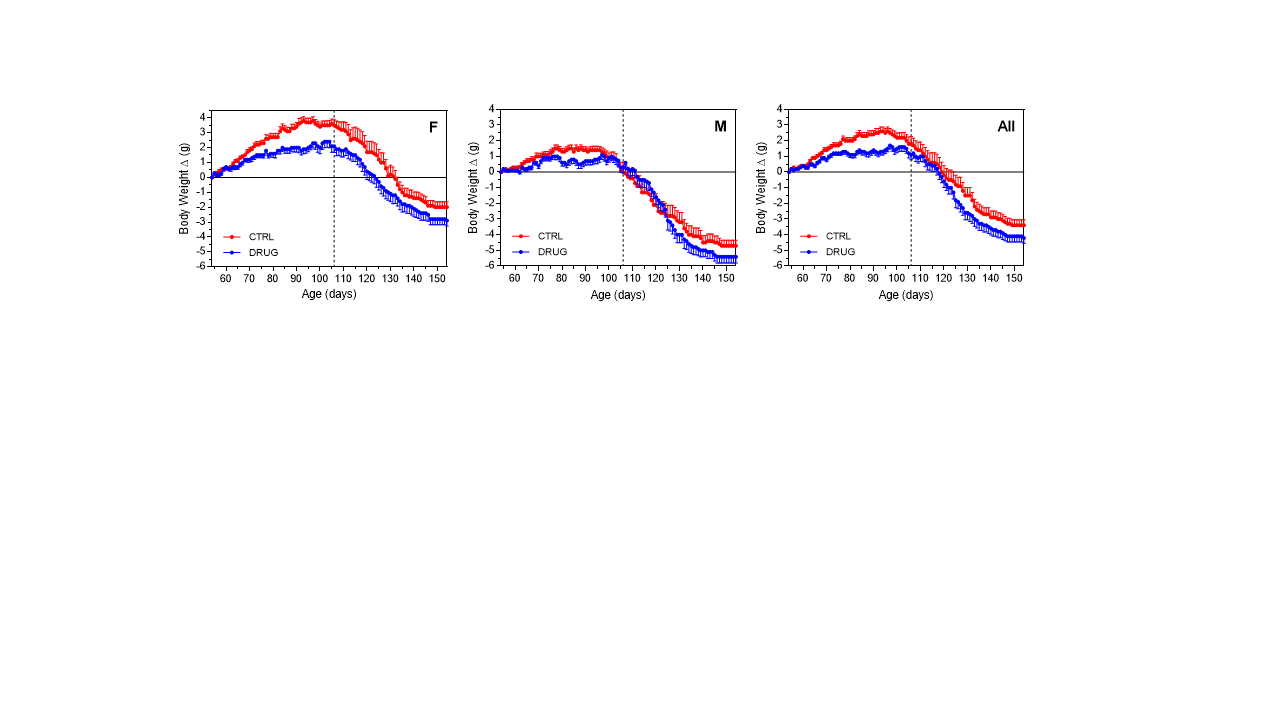

Supplement: S3 Fig — Group average body weight over time was less well maintained in both females (by as much as 1.5 g) and in males (by as much as 0.75 g) over time in GSK2606414-treated animals compared to vehicle-treated animals. Longitudinal data analysis of body weight over time using mixed-effects maximum likelihood regression with random effects for litter and for mouse, showed that GSK2606414-treated animals weighed, on average, about 0.9 g less than controls of the same age (p = 0.023). (TIF) [file pone.0292190.s003.tif]

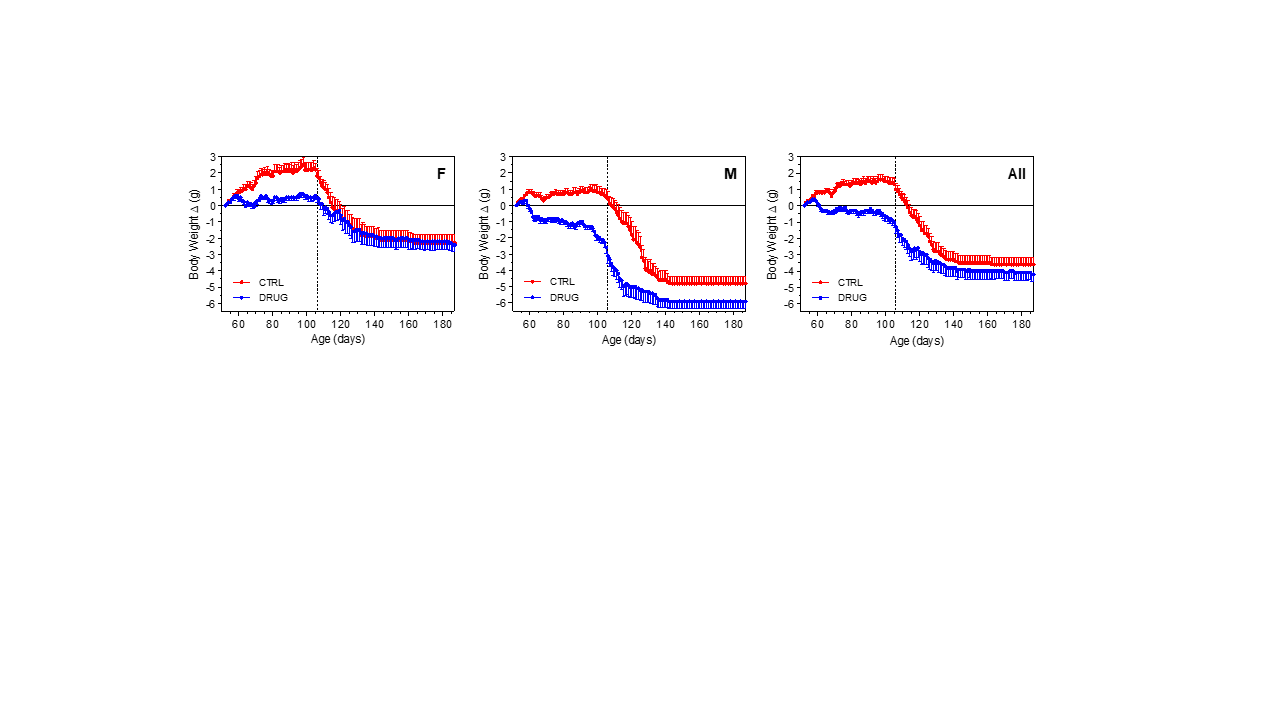

Supplement: S4 Fig — Group average body weight over time showed that GSK2606414-treated animals maintained body weight less well than corresponding vehicle control animals. At peak body weight GSK2606414-treated animals showed an ability to maintain body weight that was about 1.75 to 2 grams lighter than control when considering group average body weight over time. (TIF) [file pone.0292190.s004.tif]

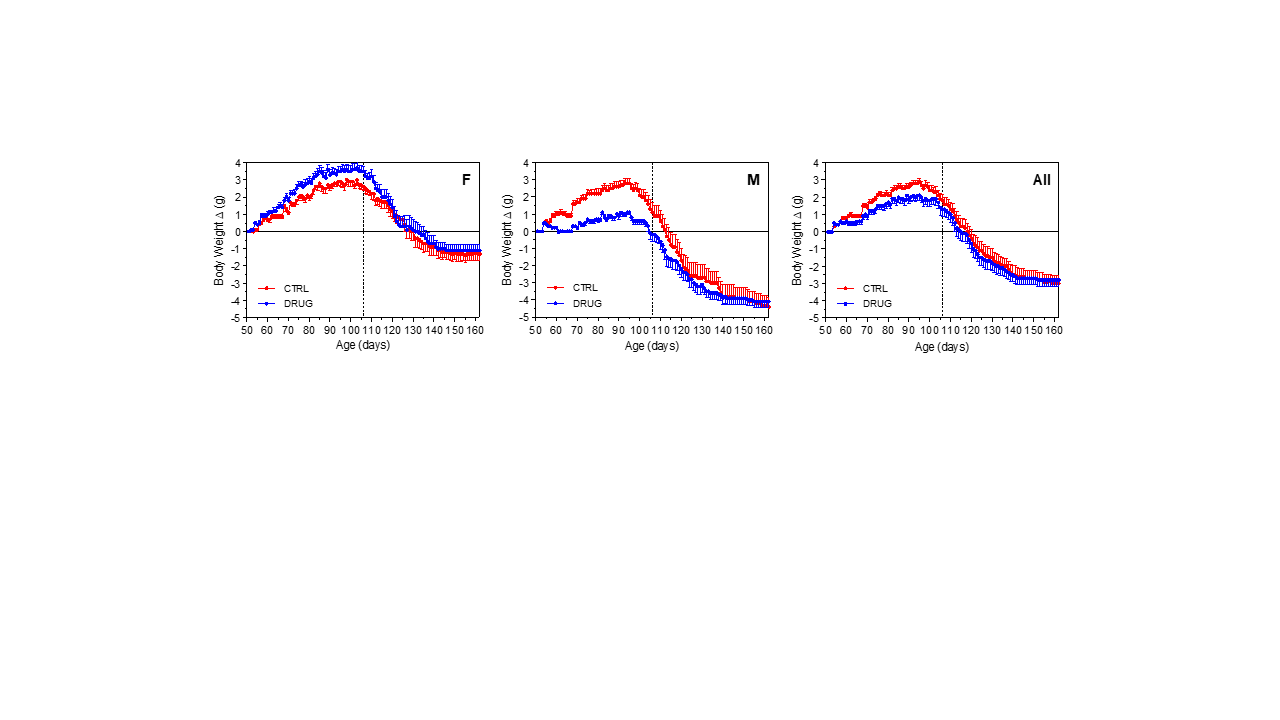

Supplement: S5 Fig — Group average body weight over time showed that female GSK2606414-treated animals maintained body weight better than corresponding vehicle control animals. At peak body weight female GSK2606414-treated animals showed an ability to maintain body weight that was about 0.5 gram heavier than control when considering group average body weight over time. Males maintained body weight less well than controls and were about 1.75 g lighter than controls over a 30-d period. Overall, there was a net reduction in body weight maintenance by about 1 g when male and female effects were combined. (TIF) [file pone.0292190.s005.tif]

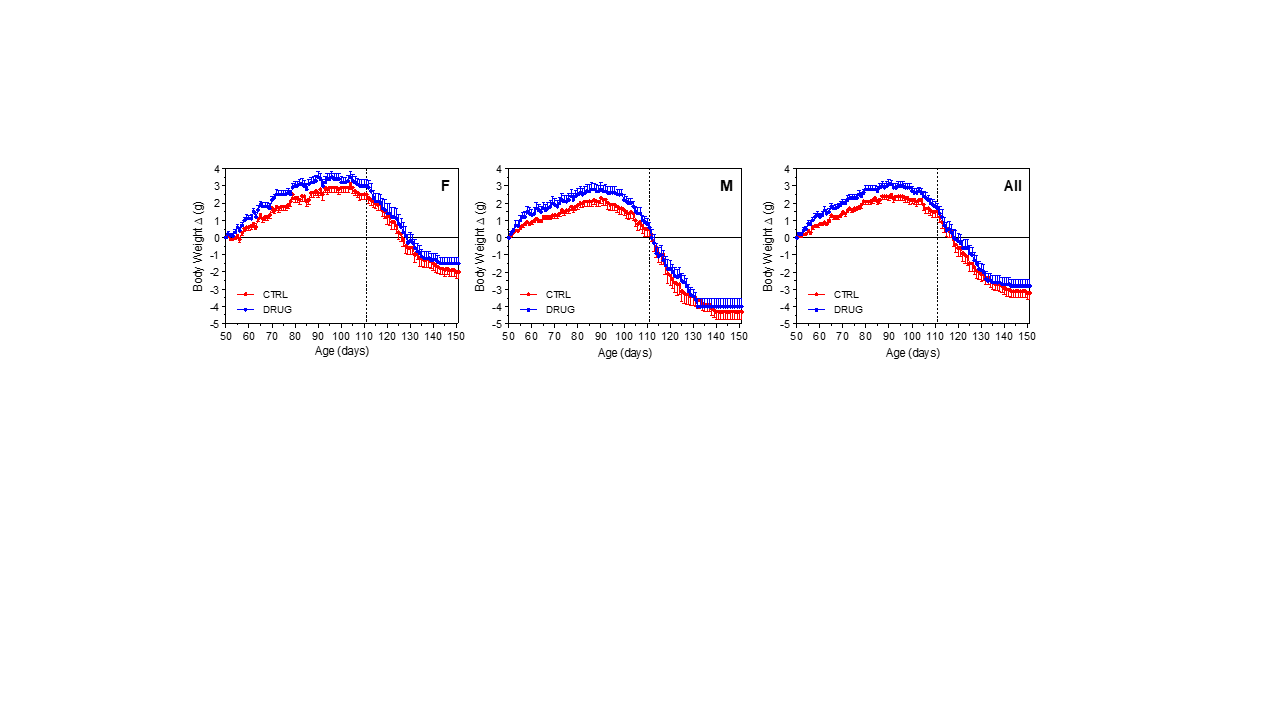

Supplement: S6 Fig — Group average body weight over time was better maintained over time in salubrinal-treated than in vehicle-treated animals. In both male and female salubrinal-treated animals body weight was about 0.5 g heavier than corresponding control animals up until the time of first death. (TIF) [file pone.0292190.s006.tif]

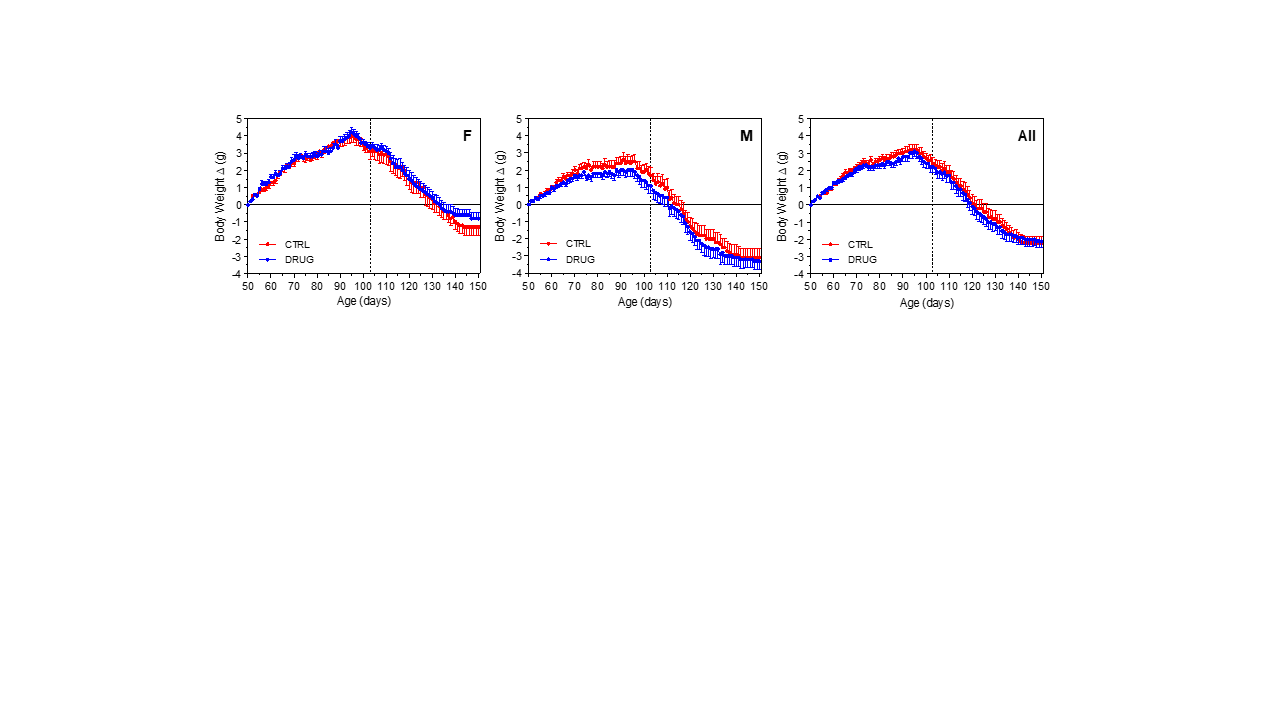

Supplement: S7 Fig — Group average body weight over time was less well maintained over time in male salubrinal-treated than in male vehicle-treated animals. In male salubrinal-treated animals body weight was about 0.5 g lighter than corresponding control animals. Female salubrinal-treated animals maintained body weight in a manner similar to vehicle controls. When males and females are combined, body weight was similar to control until about 65 days of age and then less well maintained thereafter. (TIF) [file pone.0292190.s007.tif]

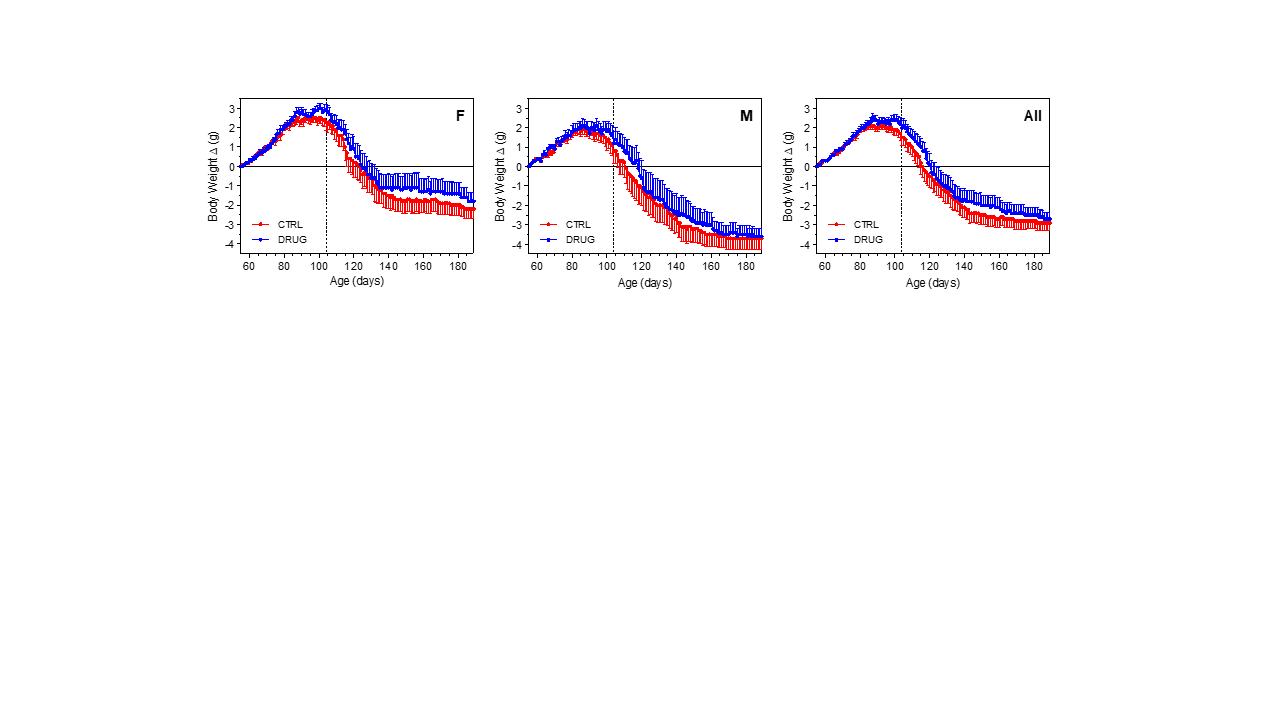

Supplement: S8 Fig — Group average body weight over time was better maintained over time in Sephin1-treated than in vehicle-treated animals from about 85 days of age (approximate time of symptom onset). Body weight was about 0.4 g heavier than in corresponding control animals. Longitudinal data analysis of body weight over time using mixed-effects maximum likelihood regression with random effects for litter, showed that sephin1-treated animals were, on average, about 0.1 g less than controls of the same age. However, this effect was not statistically significant (p = 0.86). (TIF) [file pone.0292190.s008.tif]

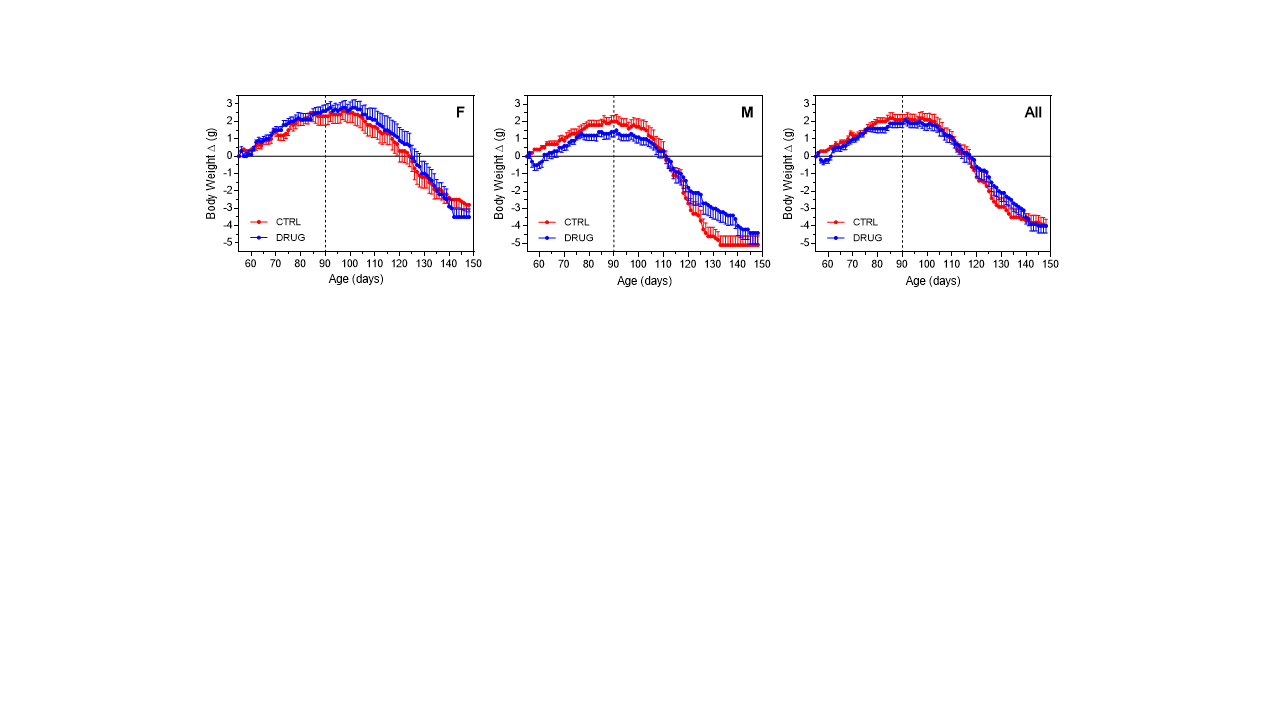

Supplement: S9 Fig — Group average body weight over time was maintained in a manner similar to, or better than, vehicle controls in sephin1-treated female animals from about 70 days of age. Body weight was as much as 0.5 g heavier than in corresponding female control animals. Male sephin1-treated animals maintained body weight less well than vehicle controls through about 105 days of age, a time well after the first animal reached its humane endpoint at age 90 days. Body weight was lighter than control by as much as 0.75 g. Longitudinal data analysis of body weight over time using mixed-effects maximum likelihood regression with random effects for litter, showed that, overall, sephin1-treated animals weighed about 0.5 g less than controls of the same age. However, this effect was not statistically significant (p = 0.24). (TIF) [file pone.0292190.s009.tif]

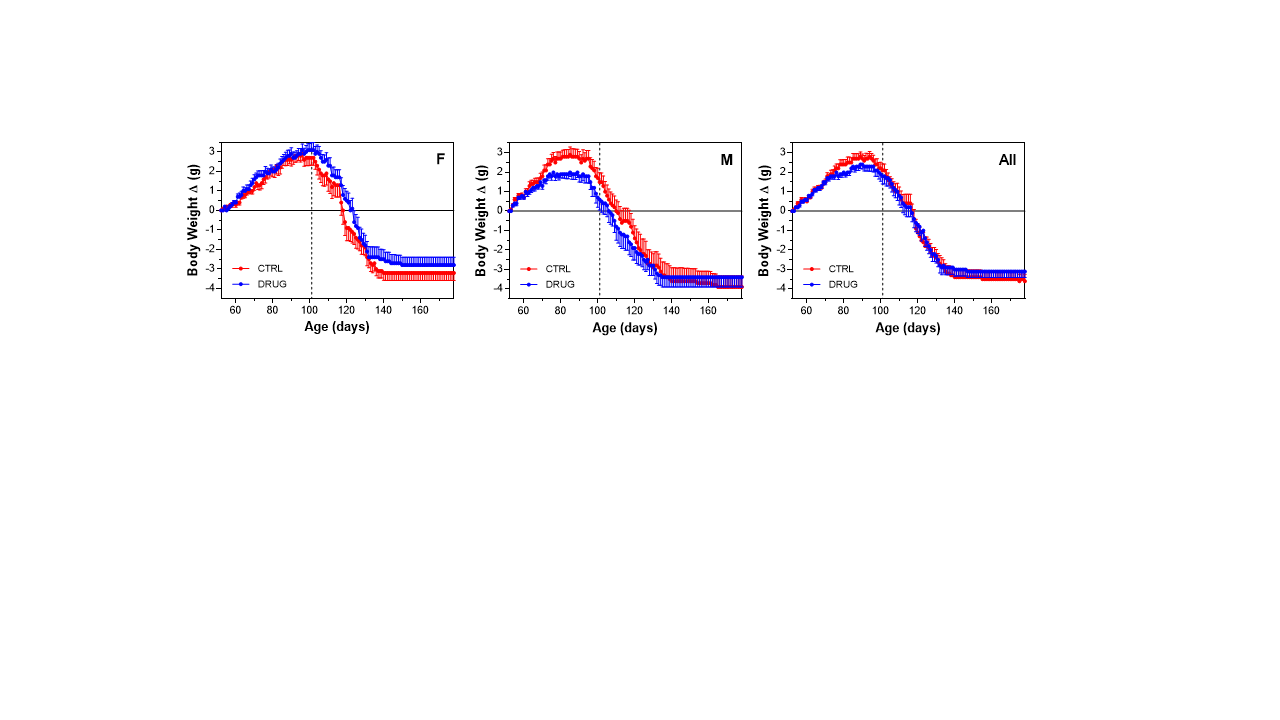

Supplement: S10 Fig — Group daily average body weight over time was better maintained (by as much as 0.5 g) over time particularly after about 90 days of age in clonidine-treated female animals compared to female vehicle-treated animals. Male clonidine-treated animals maintained body weight less well (by as much as 1 g) than male vehicle controls. When both genders were combined, clonidine-treated animals maintained body weight less well than vehicle-treated animals during the period of 70 to 100 days of age. Longitudinal data analysis of body weight over time using mixed-effects maximum likelihood regression with random effects for litter and for mouse, showed that clonidine-treated animals weighed, on average, about 0.23 g more than controls of the same age, but this effect was not statistically significant (p = 0.51). (TIF) [file pone.0292190.s010.tif]
